# Dried Vegetables as Potential Clean-Label Phosphate Substitutes in Cooked Sausage Meat

**DOI:** 10.3390/foods12101960

**Published:** 2023-05-11

**Authors:** Ingrid Weigel, Sarah Nistler, Rohtraud Pichner, Silvia Budday, Sabrina Gensberger-Reigl

**Affiliations:** 1 Food Chemistry, Department of Chemistry and Pharmacy, Faculty of Sciences, Friedrich-Alexander-Universität Erlangen-Nürnberg (FAU), Nikolaus-Fiebiger-Str. 10, 91058 Erlangen, Germany; 2 Institute of Applied Mechanics, Department of Mechanical Engineering, Faculty of Engineering, Friedrich-Alexander-Universität Erlangen-Nürnberg (FAU), Egerlandstr. 5, 91058 Erlangen, Germany; 3 Department of Nutritional, Food and Consumer Sciences, University of Applied Sciences, Leipziger Str. 123, 36037 Fulda, Germany

**Keywords:** phosphate, meat products, sausages, phosphate substitutes, clean-label, freeze-dried vegetables

## Abstract

While phosphates are key additives in sausage production, their use conflicts with consumer preferences for “natural” foods. In this study, we investigated the potential of using vegetables as “clean-label” phosphate substitutes and their effects on water holding capacity, consumer acceptance, color, softness, and tenderness. Six freeze-dried vegetables with a pH above 6.0 were added to sausage meat on a laboratory scale. Adding 1.6% freeze-dried Brussels sprouts or Red Kuri squash resulted in a similar weight gain (7.0%) as the positive control of 0.6% commercial phosphate additive. Higher vegetable concentrations (2.2–4.0%) caused a significant increase in weight (*p* ≤ 0.05, 10.4–18.4% weight gain). Similar stress was needed to compress sausages containing 1.6/4.0% Brussels sprouts (14.2/11.2 kPa) and the positive control (13.2 kPa). Indentation tests also led to similar softness results for the sausages prepared with 1.6/4.0% Brussels sprouts (15.5 kPa/16.6 kPa) and the positive control (16.5 kPa). A force of 1.25 N was needed to shear the positive control, while 1.60 N/1.30 N was needed for the samples (1.6/4% Brussels sprouts). In summary, the present study indicates that freeze-dried vegetables have the potential to effectively replace phosphate in meat products.

## 1. Introduction

Food laws regulate the use of food additives and specify maximum levels depending on the product type and application. In the European Union, the list of approved food additives in meat products includes phosphates, ascorbic acid, benzoates, and nitrates [1]. Despite their technological and sensory benefits, food additives are often perceived as negative. In particular, phosphates are associated with possible health risks. Phosphate intake through food additives has been linked to elevated phosphate serum concentrations in the general population [2]. In vitro studies and animal models have indicated that elevated phosphate serum levels might be related to vascular calcification [3,4]. In addition, a correlation between elevated serum phosphate levels and an increased risk of cardiovascular diseases has been described [5,6]. Another reason for consumer concern might be the chemical names of food additives, which are often difficult to understand [7]. Consumers perceive foods without additives as more natural and nutritious than foods containing additives [8]. Over the past years, this consumer awareness has led to notably increased sales of products advertised as free from additives or flavors [9]. Such kinds of advertisements (“free from…”, “no additives”) are called “clean-label” and describe foods that contain no additives or, at least, as little as possible. To date, however, there is no legal definition of this term [10].

In sausage production, many additives ensure the necessary technological and organoleptic characteristics and microbial safety. The reformulation of these foods is challenging because the products must meet consumer expectations as well as food quality and safety standards. Phosphates are important functional additives and cover a wide range of technical purposes. One of the most valuable effects of phosphates is improvement to the water holding capacity (WHC) of meat during processing. The water content affects the tenderness, juiciness, and the sensory attributes of meat products and is therefore a major quality parameter [11]. The WHC is influenced by several parameters such as the genetic constitution, feeding, pre- and post-slaughter treatment [12], the ionic strength [13,14,15], and the pH of the meat [16]. Phosphates improve the WHC by raising the pH above the proteins’ isoelectric point and by enhancing the ionic strength [16]. An increase in pH, however, can lead to accelerated microbiological spoilage. Thus, the pH value should not be too high to slow down the growth of spoilage agents [17].

Two different strategies have been developed to substitute or, at least, reduce phosphates in meat products: (i) the use of novel technologies such as high-pressure processing, pulsating electric field application, or ultrasonication [18,19] and (ii) the use of different functional ingredients such as modified starch or cellulose [20,21], fibers [22,23], carbonate salts [24], winter mushroom powder [25], protein hydrolysates [26], or alkaline pH solution [27]. The novel technologies are associated with high investment costs and are therefore hardly affordable for small and medium-sized butcheries. For those manufacturers, the substitution of phosphates with novel ingredients might be easier to accomplish. This concept provides an advantage for both the manufacturers and the consumers, because the avoidance of high investment costs may keep retail prices stable. Most of the available phosphate alternatives are, however, not compatible with the “clean-label” concept. Consumers value vegetables for their high level of nutrients. Using minimally processed vegetable foods to substitute typical food additives could therefore be a promising approach to meet the conditions of the “clean-label” concept as well as consumer expectations. The high nutritional value of vegetables is primarily determined by vitamins, minerals, dietary fibers, and aromatic compounds. Additionally, vegetables contain nitrogen compounds such as proteins and free amino acids; carbohydrates such as mono-, oligo-, and polysaccharides; organic acids such as citric or maleic acid; and other specific components such as pigments (e.g., chlorophyll) or glucosinolates [28]. Some vegetables can also be a source of phosphates. For example, Brussels sprouts contain up to 86 mg of phosphor per 100 g [29]. Nevertheless, to the best of our knowledge, vegetables have not yet been used as phosphate substitutes, with one exception. Choe et al. replaced phosphate in sausage meat with winter mushroom powder [25].

In this study, we investigated the potential of using freeze-dried vegetable foods as a “clean-label” phosphate substitute in cooked sausage meat with the goal of replacing phosphate as an ingredient. To evaluate the possible disadvantages of vegetable-based phosphate substitutes in cooked sausage meat, the physical parameters of WHC, color, softness, and tenderness of the samples and consumer acceptance were analyzed.

## 2. Materials and Methods

### 2.1. Overview over the Study and Selection Criteria

**Figure 1:**
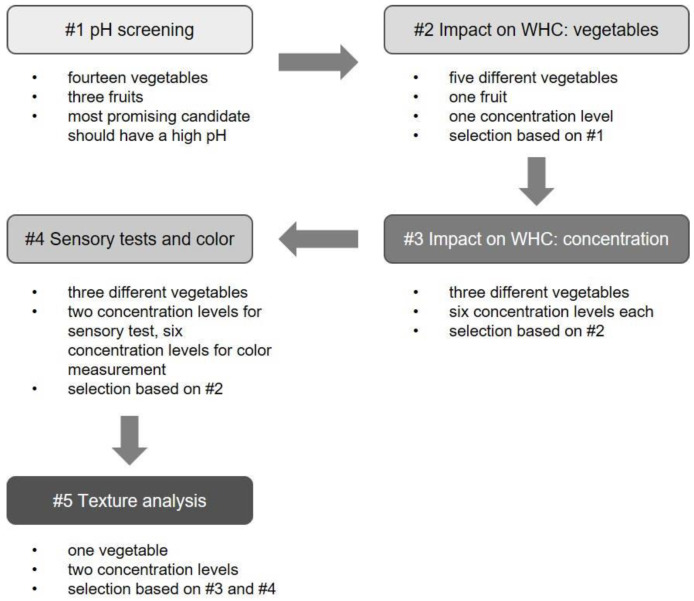
Overview over the study and selection criteria for the different experiments.

Figure 1 gives an overview over the whole study. At the beginning, the pH values of fourteen vegetables and three fruits were screened (Figure 1 #1). Based on this step, the six vegetables with the highest pH values were selected for further experiments (Figure 1 #2). The three vegetables with the highest impact on WHC were selected for concentration-dependent measurements (Figure 1 #3) and for color assessment and sensory tests (Figure 1 #4). Finally, the most promising vegetable from the preceding experiments #3 and #4 (Figure 1) was subjected to texture analysis.

### 2.2. Meat, Plant Products, and Reagents

Pork meat and vegetable foods (avocado, beetroot, broccoli, Brussels sprouts, cantaloupe, cassava, celery root, date, papaya, peas, Red Kuri squash, savoy cabbage, spinach, sweet corn, sweet potato, white cabbage, and zucchini) were purchased from a local retailer. The produce used for the experiments was fresh, except for frozen Brussels sprouts, peas, and spinach and dried dates. All fruits and vegetables were unprocessed and free of additives. The kernels of Red Kuri squash were removed and corn kernels were separated from the cobs prior to use. The commercial phosphate additive for sausage production (“Perfektin,” trisodium diphosphate and pentasodium triphosphate, Moguntia Food Group, Mainz, Germany) was a generous gift from Rehm’s Hofladen (Denkendorf, Germany). Sodium chloride was purchased from Sigma Aldrich (Taufkirchen, Germany).

For the sensory tests, dried Brussels sprouts and sweet corn were purchased from Fuster Gefriergetrocknet (Appenzell Eggerstanden, Switzerland). Dried Red Kuri squash was obtained from the company Trokost Gemüsetrocknungs- und Nahrungsmittel (Sandhausen, Germany).

### 2.3. pH Screening of Vegetable Foods

All vegetable foods were cut into pieces (not larger than 3 × 3 × 3 cm^3^), if necessary, and minced at 2500 rpm for 3 × 10 s using a knife mill (GM 200, Retsch, Haan, Germany). The pH of the products was analyzed electrochemically using a pH electrode. For this purpose, each homogenate was mixed with the same weight proportion of ultra-pure water.

### 2.4. Preparation of Minced Meat and Vegetable Additives

Pork meat without residual fat and tendon tissue was cut into pieces of 3 × 3 × 3 cm^3^ and minced in a Retsch knife mill GM 200 at 2500 rpm for 3 × 10 s to achieve a homogenous product. Aliquots of the minced meat were kept at −20 °C and thawed at 4 °C immediately prior to use. Avocado, Brussels sprouts, peas, Red Kuri squash, spinach, and sweet corn were minced as described in Section 2.3. After freezing at −80 °C, the vegetables were freeze-dried for 4 days. Thermal energy was supplied via contact with an ambient temperature of 22 °C to initiate and support the sublimation process. The drying vacuum was set to 5 mbar (Savant Novolyphe NL 150, Thermo Fisher Scientific, Schwerte, Germany). The dry vegetables were crushed with a spatula. For experiments on concentration dependency, the dry substances were further comminuted using a T18 Ultra Turrax (IKA, Staufen, Germany).

### 2.5. Preparation of Sausage Meat Samples

To prepare the sausage meat samples on a laboratory scale, aliquots of 5 g of minced meat samples (tempered to 4 °C) were weighed into reaction tubes (Greiner, Frickenhausen, Germany) and added to 1 mL of sodium chloride in water (50 mg/mL). The samples were mixed with 4 mL of ice-cold water and 200 mg each of the freeze-dried vegetables, respectively. The experiments on concentration dependency were carried out with decreasing concentrations of the freeze-dried substances (200/170/140/110/80/50 mg of the lyophilizates). Controls were prepared independently for each series of experiments by adding either 1 mL of commercial phosphates in water (30 mg/mL) and 3 mL of ice-cold water (positive control) or 4 mL of ice-cold water (negative control). A table showing detailed sample formulations is available in the Supplementary Materials Table S1. All samples were thoroughly mixed for 1 min using a spatula, followed by a dwell time of 30 min at 4 °C. The samples were simmered in a water bath at 85 °C for 90 min and cooled to room temperature (RT) using ice-cold water before centrifugation at 1840× *g* for 10 min at RT (Rotofix centrifuge 320, Hettich, Tuttlingen, Germany). The supernatant was decanted and collected. The pH value of the supernatant was monitored electrochemically using a pH electrode. All samples had a cylindrical shape of approx. 20 mm diameter and approx. 20 mm height. The samples prepared in this way were used for all tests except for the sensory evaluation (see Section 2.7).

### 2.6. Determination of Water Holding Capacity

The total weight of the samples containing minced meat and one of the additives was determined before adding water. After preparing the sausage meat samples as described in 2.5, unbound residual water was drained off by placing the sample tubes upside down on a paper towel for 10 min. The processed samples were weighed again and the WHC was expressed as the weight gain in percent. The difference was calculated as follows:


(1) $$\frac{mass\;(after\;treatment)-mass\;(before\;treatment)}{mass\;(before\;treatment)}*100\%$$

To focus on the effect of the vegetables and to consider the variability of the different meat batches, all values were corrected by the weight gain determined for the negative control. Each sample was analyzed in duplicates on one day. The experiments were repeated on three different days resulting in six measurements per sample. Results are reported as mean values ± standard deviation.

### 2.7. Sensory Tests

Samples were prepared as described in Section 2.4 and Section 2.5 with slight modifications. To ensure that all panelists received identical samples, the scale of the experiment was increased. Additionally, kitchen appliances were used to prepare the samples instead of laboratory equipment to guarantee panelist safety and adherence to ethical standards. The pork meat was cut into pieces not larger than 3 × 3 × 3 cm^3^. Meat (350 g), water, salt, as well as phosphate (positive control) or the dried vegetables were placed into a kitchen mill (Thermomix TM5, Vorwerk, Wuppertal, Germany) and minced for 20 s at its maximum speed to achieve homogenous sausage meat. The final concentrations of the vegetables were 4.0 or 1.6% (*w*/*w*) Brussels sprouts, Red Kuri squash, or sweet corn. The sausage meat was filled into an artificial sausage skin (caliber 19 mm) and simmered at 85 °C for 90 min. The cooked sausage meat was cooled to RT in an ice-cold water bath. The skin was removed and the samples were cut into pieces of 7 g, which were halved.

The samples were subjected to a sensory test with 23 untrained volunteers (median age: 31 years, range: 22 to 61 years; 15 females, 8 males) consisting of students and employees of the FAU Department of Chemistry and Pharmacy. Written informed consent was obtained from all volunteers. The study was approved by the Ethics Commission of Friedrich-Alexander-Universität Erlangen-Nürnberg (log 22-213-ANF). Water and low-salt crackers were offered to the panelists to cleanse the palate. The samples were provided in a random order with a dummy sample to reduce first-order effects [30]. As the dummy sample, the negative control (see Section 2.5) was used. The volunteers on the sensory panel were asked to indicate their score of acceptance. Testing was performed with the 11-point hedonic scale with labelled affective magnitude (LAM) anchors from Schutz and Cardello [30,31]. The attributes and the corresponding values are shown in Supplementary Materials Table S2. The average LAM value was calculated for each sample and statistical differences were evaluated (see Section 2.10). In this study, the 11-point hedonic scale with LAM was favored, since Greene et al. detected more significant differences on the LAM scale compared to the 9-point hedonic scale when the samples were scored around the neutral point [32]. As we expected scoring of the majority of the samples around the neutral point, we decided to use the eleven-point scale with LAM instead of the 9-point hedonic scale, although some studies indicate that the scales perform similarly [31,33].

### 2.8. Color Assessment

The color of the sausage meat samples (see Section 2.5) was measured using a portable sphere-type spectrophotometer (CM-700d, Konica Minolta, Stockstadt, Germany) and expressed in L*, a*, and b* color space with L* indicating lightness, a* the red/green coordinate, and b* the yellow/blue coordinate. Samples were prepared in triplicates. Three replicates for each sample were determined at the top, the bottom, and the lateral surface. Results are reported as mean values ± standard deviation.

### 2.9. Assessment of Textural Characteristics

**Figure 2:**
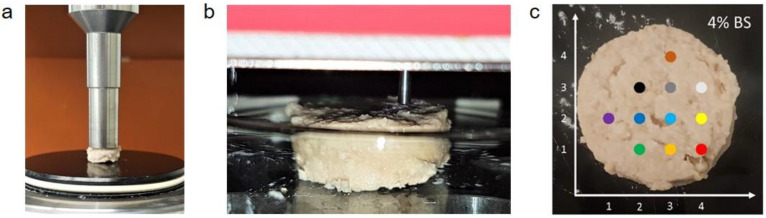
(**a**) Sample in a Discovery HR-3 Rheometer prior to the compression test; (**b**) sample in testing device before indentation; (**c**) distribution of the investigated indents, each marked as a colored dot; *x*- and *y*-axes in mm.

For the assessment of the textural characteristics, the samples described in Section 2.5 were used. For compression and indentation measurements, six sausage meat samples were prepared with 4.0 or 1.6% (*w*/*w*) freeze-dried vegetables or 0.6% (*w*/*w*) phosphates as a positive control, respectively. The sausage meat samples were cut into slices of 5 mm thickness. Six cylindrical cut-outs from different areas of these slices (cut from the center towards the edges, each 8 mm in diameter) were subjected to a compression test (Discovery HR-3 Rheometer, TA Instruments, New Castle, Delaware, USA, Figure 2a). The compressibility was set to 50% of the initial sample height and three cycles with a speed of 100 µm/s were applied. The maximum stress was measured in kilopascals (kPa) and is presented as the mean ± standard deviation of the six samples.

Additionally, the sausage meat slices were analyzed using indentation (ZHN-Universal Nanomechanical Testing System, Zwick, Ulm, Germany, Figure 2b). A grind pattern with a distance of 4 mm was used to choose up to eleven indents (Figure 2c). The indentation test was conducted with a 1.8 mm cylindrical flat-punch indenter tip and a crosshead speed of 5 µm/s to a depth of 50 µm (Figure 2b). The maximum force (unit: mN) necessary to penetrate the sausage meat samples was measured (Supplementary Materials, Figure S1a). The analysis process involved three phases: loading, holding, and unloading (Supplementary Materials, Figure S1a). The contact stiffness *k* of each indent was defined as the slope of the loading phase. The slope was calculated via linear regression (Supplementary Materials, Figure S1b). With *d* as the diameter of the indenter tip (*d* = 1.8 mm), the effective modulus *E* was then calculated according to the equation

(2) $$E_{eff}=\frac{3}{4}\frac{k}{d}$$

and quantifies the samples’ resistance to being deformed [34].

The Warner–Bratzler shear force (unit: N) was measured according to Uzlaşır et al. with minor modifications after optimization of the test setup [35]. Eight samples containing 4.0% (*w*/*w*) freeze-dried Brussels sprouts, eight samples containing 1.6% (*w*/*w*) freeze-dried Brussels sprouts, and seven samples containing 0.6% (*w*/*w*) phosphates as a positive control were analyzed. All samples were cut into 10 mm thick slices prior to measurement. A texture analyzer Z 1.0 (Zwick Roell, Ulm, Germany) equipped with a force transducer type KAF-TC and a v-notch Warner–Bratzler blade (114 mm × 70 mm × 3 mm) was used. The blade moved at a rate of 200 mm/min. The maximum and the average force necessary to shear the samples were evaluated within a measuring distance of 20 mm.

### 2.10. Statistical Analysis

Microsoft Excel 2019 and MATLAB R2021a (first release of MATLAB in 2021) were used for statistical analysis. The results were depicted as the mean ± standard deviation. The results were compared to the positive control using a one-tailed Student’s *t*-test for unpaired samples. Homoscedasticity was tested via F-tests in advance. The levels of significance were * *p* ≤ 0.05; ** *p* ≤ 0.01; and *** *p* ≤ 0.001.

## 3. Results and Discussion

### 3.1. pH Screening in Vegetable Foods

The WHC of meat is influenced, among other parameters, by the pH value. The idea that fruits or vegetables with high pH values can replace phosphates in sausage is based on the acid–base concept. A compound with a basic pH can neutralize a compound with an acidic pH. Since meat is slightly acidic, a compound with a high pH value is needed to increase the pH in meat. We screened for vegetables with higher pH values that might be able to buffer the slightly acidic pH of meat so as to increase the pH of the sausage meat and, consequently, improve the WHC. The goal of our experiment was to identify six promising candidates whose potential as phosphate substitutes would be further investigated. Since no comprehensive database is available for pH values in food, we screened the pH in 14 different randomly selected vegetables and three fruits. The main focus was, however, on vegetables, because most fruits are acidic. The pH values were measured in duplicate and ranged from a pH of 5.45 to a pH of 7.25 (Table 1). The range of the duplicate determinations was less than or equal to 0.02. The highest values were observed for peas, followed by spinach and Red Kuri squash (all > pH 6.90), while sweet potato, date, and papaya showed the lowest pH values (all < pH 5.90). Vegetable additives with a high pH might be promising options for increasing the pH in sausage meat, and thus increasing the WHC. The six vegetable foods with the highest pH values (peas, spinach, Red Kuri squash, sweet corn, avocado, and Brussels sprouts) were considered for further investigation, with one exception. Although zucchini had a slightly higher pH value, Brussels sprouts were chosen for further experiments. Zucchini belongs to the same plant family as Red Kuri squash, while Brussels sprouts belong to *Brassicaceae*, and thus represent another plant family. To expand the number of selected plant families, Brussels sprouts instead of zucchini were included in the following analyses. The phosphate content of the vegetable products had no influence on the decision which products were investigated further.

### 3.2. Effect of Different Additives on the WHC of Sausage Meat Samples

**Figure 3:**
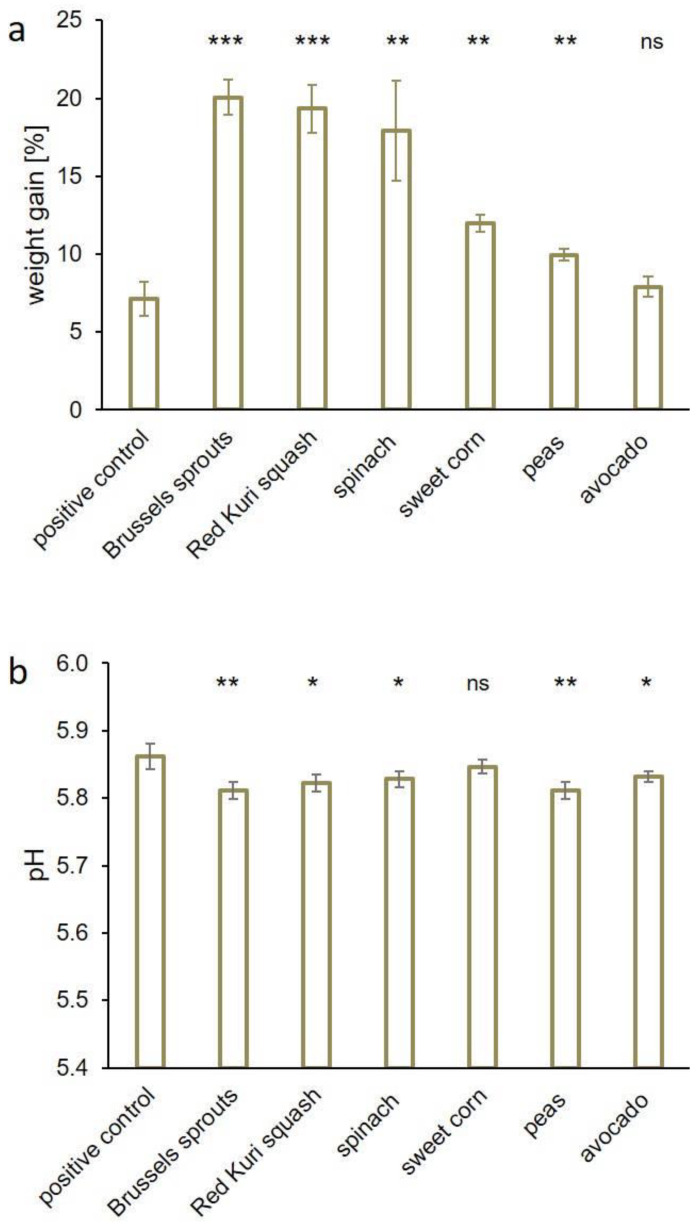
(**a**) WHC of sausage meat samples containing 4% (*w*/*w*) freeze-dried Brussels sprouts, Red Kuri squash, spinach, sweet corn, peas, or avocado. The positive control contained a commercial phosphate food additive at a concentration of 0.6% (*w*/*w*). The WHC is expressed as the weight gain in percent. (**b**) The corresponding pH values of the samples are displayed (mean ± standard deviation). Statistically significant differences in the weight gain or the pH value, respectively, between the positive control and the samples were evaluated. The levels of significance were * *p* ≤ 0.05; ** *p* ≤ 0.01; *** *p* ≤ 0.001; and not significant (ns) *p* > 0.05.

The addition of Brussels sprouts (4% *w*/*w*) led to a weight gain of 20.1%, followed by Red Kuri squash (+19.3%), spinach (+17.9%), sweet corn (+12.0%), peas (+10.0%), and avocado (+7.9%). Compared to the positive control (+7.1%), all vegetable additives except avocado caused significant weight gain due to an improved WHC (Figure 3a). The pH value of all the samples was above a pH of 5.80 (Brussels sprouts and peas: pH 5.81; Red Kuri squash: pH 5.82; avocado and spinach: pH 5.83; sweet corn: pH 5.85; Figure 3b). The positive control had a pH value of 5.86, while the negative control had a pH value of 5.78. Thus, all six vegetables increased the WHC of the sausage meat samples significantly, with Brussels sprouts, Red Kuri squash, and spinach leading to the greatest increases. In samples prepared with these additives, the weight gain exceeded the weight gain of the positive control by a factor of three. The addition of sweet corn or peas increased the weight by a factor of 1.7 and 1.4, respectively, whereas avocado samples showed similar results as that of the positive control. One reason for this could be the composition of avocado, which contains more fat than the other vegetable foods used. All additives caused a pH increase in the samples, which was not, however, directly linked to the gain in weight. Interestingly, the positive control had the highest pH value, but not to the highest increase in weight. Brussels sprouts and sweet corn contain high amounts of amino acids such as aspartic or glutamic acid, arginine, lysine, or histidine [29], which can have buffering effects. This observation indicates that the pH value was not the most important factor influencing the weight. The rehydration (swelling, uptake, or retention) of the freeze-dried vegetables may also play an important role in holding water and may contribute to weight gain. Plant fibers could be another factor influencing the WHC. Previous studies reported an improved WHC in meat products when dietary fibers were added [36,37]. The interaction between vegetable fibers and meat fibers might also contribute to the improved WHC. It has been shown in silico that hydrogen bonds between dietary fiber and meat proteins like actin or myosin can be formed [38]. The hydrogen bonds can stabilize the fiber complexes so that water molecules can be embedded between the elongated fibers. Additionally, other factors such as enhanced ionic strength or calcium-complexation caused by vegetable components may also contribute to an improved WHC.

**Figure 4:**
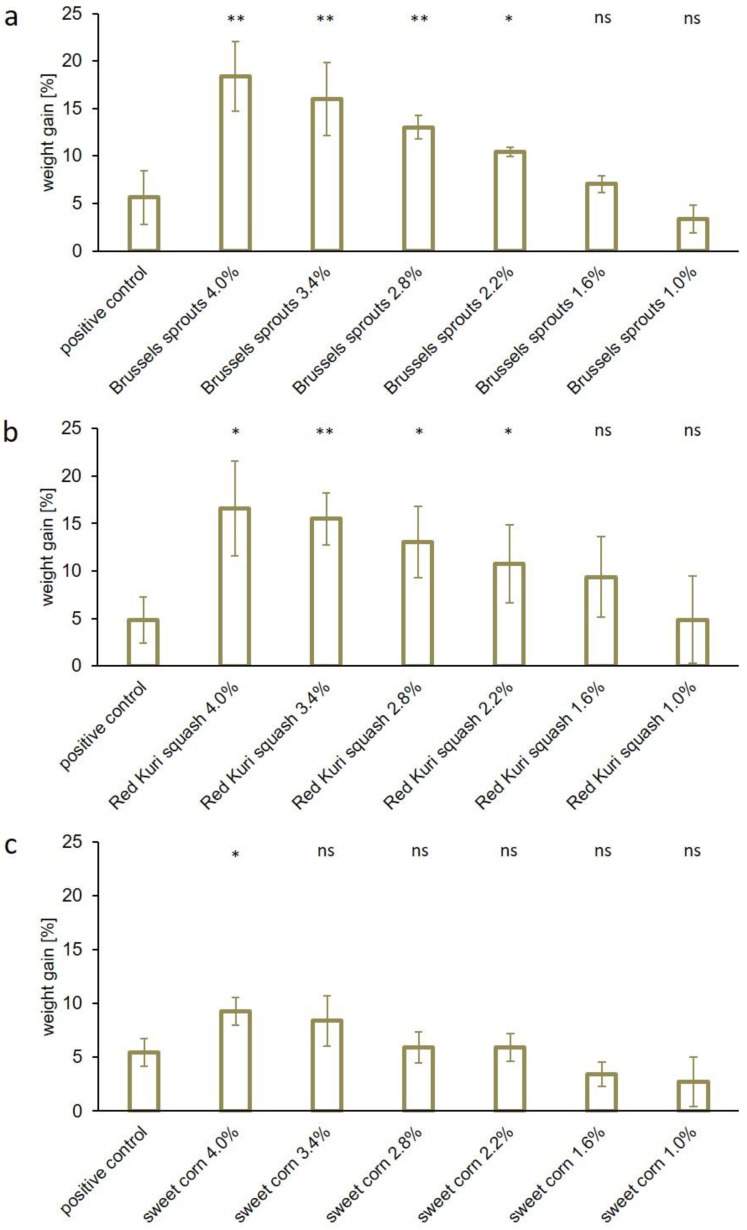
WHC of sausage meat samples containing 4–1% (*w*/*w*) freeze-dried (**a**) Brussels sprouts, (**b**) Red Kuri squash, or (**c**) sweet corn. The positive control contained 0.6% (*w*/*w*) commercial phosphates. The WHC is expressed as the weight gain in percent (mean ± standard deviation). Statistically significant differences between the positive control and the samples were evaluated. The levels of significance were * *p* ≤ 0.05; ** *p* ≤ 0.01; and not significant (ns) *p* > 0.05.

The use of spinach caused a strong discoloration, so Brussels sprouts, Red Kuri squash, and sweet corn seemed to be the most promising phosphate alternatives. Thus, we investigated the concentration dependency of these additives. For that purpose, sausage meat samples were prepared with six different concentrations of freeze-dried vegetables and the WHC of the samples was determined. Figure 4 presents the concentration-dependent effects of the added dry vegetables on the WHC compared to the respective positive controls. The use of 4.0/3.4/2.8/2.2/1.6 or 1.0% (*w*/*w*) Brussels sprouts (Figure 4a) caused a weight gain of 18.4/16.0/13.0/10.4/7.0 or 3.3% in the sausage meat samples (vs. 5.6% observed for the positive control). The WHC also varied depending on the amount (4.0/3.4/2.8/2.2/1.6 or 1.0% (*w*/*w*)) of Red Kuri squash and resulted in a 16.6/15.5/13.0/10.8/9.4 or 4.9% weight gain, respectively, while 4.8% was observed in this series for the positive control (Figure 4b). The use of 4.0/3.4/2.8/2.2/1.6 or 1.0% (*w*/*w*) sweet corn led to a weight gain of 9.3/8.4/5.9/5.9/3.4 or 2.7%, respectively, compared to a 5.5% weight gain in the positive control (Figure 4c). Each freeze-dried vegetable induced a concentration-dependent increase in the WHC, but only moderate effects were found when sweet corn was added. In a concentration of 2.2% (*w*/*w*), sweet corn resulted in a weight gain comparable to the positive control. The lowest concentration of 1.0% (*w*/*w*) showed only a slight positive effect on the WHC, which was less than the gain in the positive control. Results comparable to those of phosphates were obtained with concentrations of 1.0% (*w*/*w*) Red Kuri squash and 1.0–1.6% (*w*/*w*) Brussels sprouts. These results are in line with a study by Choe et al., who efficiently replaced phosphates with 2% lyophilized winter mushroom powder [25].

The pH values of the tested meat samples were 5.80–5.81 (Brussels sprouts), 5.80–5.82 (Red Kuri squash), and 5.80–5.84 (sweet corn). The pH values of the positive and the negative controls were 5.88 and 5.78, respectively (Supplementary Materials, Table S3). The pH values of the vegetable-containing samples were all lower than the pH of the positive control and ranged only slightly above the pH of the negative control. Choe et al. also observed no significant differences in pH when substituting phosphates with >1% winter mushroom powder [25]. A reason for the lower pH might be the buffering effect of proteins or other ingredients in the freeze-dried vegetables. It can be assumed that the effects on the WHC caused by the vegetable additives are not predominantly mediated by altering the pH in sausage meat samples. Supposedly, rehydration and the interaction of vegetable components with the meat fibers are major factors in this context. Besides, vegetables can be a source of phosphate, which may positively influence the WHC of the sausage meat. For example, Brussels sprouts contain up to 86 mg of phosphor per 100 g [29]. Thus, the addition of 4% Brussels sprouts supplemented the samples with up to 688 µg of phosphor, considering a dry matter of 25% [29]. This amount is too low to improve the WHC of the samples significantly. However, the detailed mechanisms how the vegetable additives used in this study interact with the meat fibers are not known yet. Follow-up studies are required to gain further insights.

### 3.3. Sensory Tests

The overall acceptability of sausage samples containing sweet corn was not significantly different compared to the samples prepared with phosphate. Sausage meat containing Brussels sprouts or Red Kuri squash, however, received significantly lower acceptance values than the positive control (Table 2). Notably, the ratings of all the samples show a quite broad distribution from “dislike very much” (equals LAM 22.25) to “like very much” (equals LAM 78.06) or even “like extremely” (equals LAM 87.11; 4.0% Red Kuri squash), respectively. The average LAM values of all samples vary around the value of 50, which equals “neither like nor dislike”. The reason for this effect could be that the tested intermediate product did not contain any fat or spices except salt. Besides, bitter tasting glucosinolates and their hydrolysis products such as goitrin might lead to a distinct taste in samples containing Brussels sprouts [39], and thus might slightly reduce consumer acceptance. It has been shown that the bitter taste of goitrin and other bitter compounds can be masked in liquid samples [40]. Such strategies should be considered in the development of new products and recipes.

### 3.4. Color and Texture of the Sausage Meat Samples

The color of the sausage meat samples prepared with vegetable additives and with phosphate as the positive control was measured spectrophotometrically. The results are displayed in Table 3. The sample color varied depending on the additives and on the concentrations. The values for lightness, red/green, and yellow/blue coordinates in the samples with Brussels sprouts ranged as follows: L*: 75.73–76.80, a*: 1.27–1.68, and b*: 19.37–17.01. Preparation with Red Kuri squash resulted in L* 75.50–76.60, a* 4.03–2.50, and b* 24.80–19.17, and addition of sweet corn in L* 77.06–75.86, a* 2.71–2.10, and b* 21.12–17.33. The positive control showed L* 78.91, a* 0.87, and b* 17.29. The values for lightness and red/green coordinates were influenced significantly at all concentration levels investigated. The values for yellow/blue coordinates were significantly increased in samples with 2.2% and higher contents of Brussels sprouts or sweet corn and in samples with Red Kuri squash concentrations ≥1.6%.

All sausage meat samples containing vegetables showed a significantly lower L* value than the positive control, indicating that the samples were slightly darker. The a* and b* values of all test samples were higher than those of the positive control, which means that the samples were more reddish and less yellow than the phosphate-containing control. In particular, samples containing Red Kuri squash showed an increase in the proportion of red (Δa*−3.16 to −1.63) and blue (Δb* −7.51 to −1.88), which was most likely due to the vegetable color. In samples containing Brussels sprouts or sweet corn, the proportion of blue increased also, but only at concentrations of 4.0% (*w*/*w*; Brussels sprouts) and 2.8% (*w*/*w*; sweet corn). The red content was also higher in these samples compared to the positive control. The data show direct color effects of the added vegetables. The distinct differences are, however, rather small and do not compromise the product quality. The color of the samples prepared with Brussels sprouts was the most similar to the positive control.

In total, three different parameters were investigated to characterize the mechanical properties of the meat sausage samples: compression, indentation, and Warner–Bratzler shear force. We used these three different indicators to obtain a broad description of the texture and to ensure that possible differences between the positive control and the samples were recognized. Sausage meat samples were prepared with 1.6 or 4.0% (*w*/*w*) Brussels sprouts or 0.6% (*w*/*w*) commercial phosphates and subjected to these three measures. Brussels sprouts were preferred for these experiments because of their higher WHC compared to sweet corn, their lower variation compared to Red Kuri squash, and their higher similarity in color compared to the positive control. A concentration of 4.0% Brussels sprouts was chosen because it led to the highest WHC, while the concentration of 1.6% Brussels sprouts did not result in a significantly different WHC, and thus to the most similar results to phosphate. Based on these experiments, the softness and tenderness of the samples were compared to the characteristics of the positive control.

**Figure 5:**
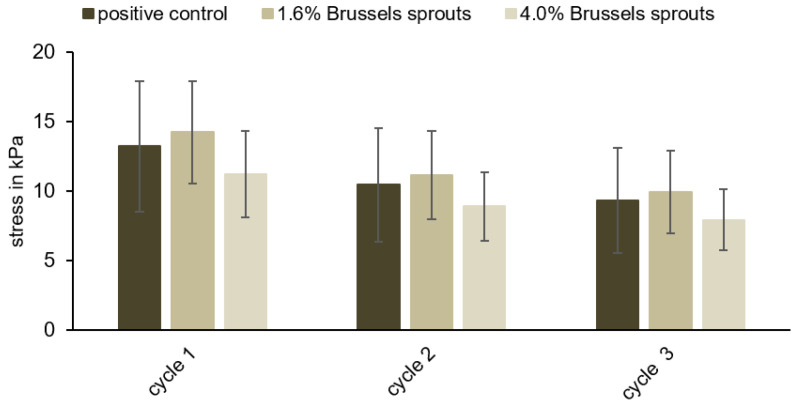
Results of the compression tests for the positive control (0.6% (*w*/*w*) commercial phosphates) and samples containing 1.6 or 4.0% (*w*/*w*) Brussels sprouts, respectively. No significant differences between the positive control and the samples were observed (*p* > 0.05).

An average stress of 11.22 ± 3.11 kPa (4.0% (*w*/*w*) Brussels sprouts) and 14.23 ± 3.67 kPa (1.6% (*w*/*w*) Brussel sprouts) was necessary during the first cycle to compress the samples to 50%, compared to 13.24 ± 4.69 kPa for the positive control (Figure 5). Two more compression cycles were measured to identify possible differences between the samples and the positive controls (Figure 5; Supplementary Materials, Figure S2), but the compression behaviors of the sausage meat samples containing 4.0 or 1.6% (*w*/*w*) Brussels sprouts and the positive control were very similar. No significant differences were observed in terms of the stress required to compress the samples (*p* > 0.05).

**Figure 6:**
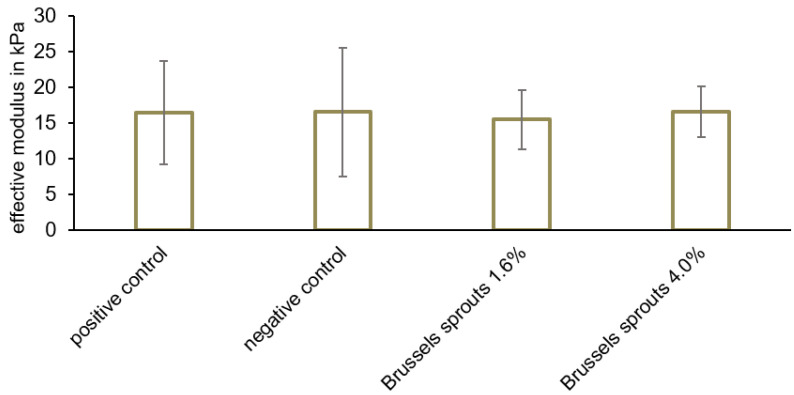
Effective modulus of the samples containing 0.6% (*w*/*w*) commercial phosphates (positive control), or 1.6 or 4% (*w*/*w*) Brussels sprouts, respectively (mean ± standard deviation). The effective modulus did not differ significantly (*p* > 0.05).

Indentation tests resulted in effective moduli of 16.48 ± 7.24 kPa (positive control), 15.45 ± 4.15 kPa (1.6% (*w*/*w*) Brussels sprouts), and 16.58 ± 3.57 kPa (4% (*w*/*w*) Brussel sprouts; Figure 6). The positive control and the samples did not differ significantly in effective modulus (*p* > 0.05), indicating that there are no differences in softness between the positive control and the sausage meat samples containing freeze-dried Brussels sprouts.

**Figure 7:**
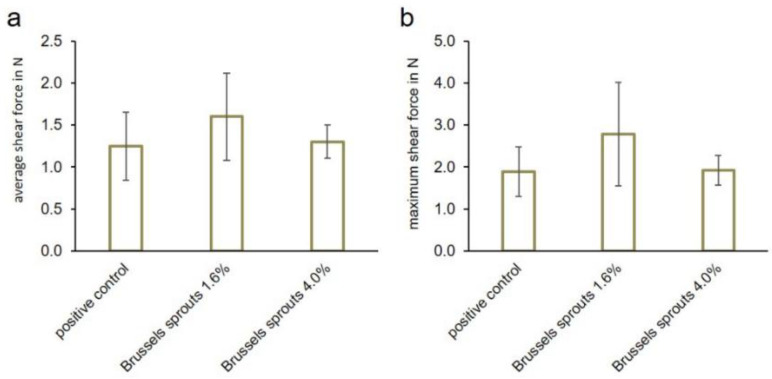
(**a**) Average shear forces and (**b**) maximum shear forces of the samples containing 0.6% (*w*/*w*) commercial phosphates (positive control) or 1.6 or 4% (*w*/*w*) Brussels sprouts, respectively (mean ± standard deviation). The shear forces did not differ significantly (*p* > 0.05).

Warner–Bratzler shear force tests resulted in 1.25 ± 0.40 N (average force) and 1.89 ± 0.59 N (maximum force) for the positive control. Forces of 1.60 ± 0.52 N (average force) and 2.78 ± 1.23 N (maximum force) were needed to shear the samples containing 1.6% (*w*/*w*) Brussels sprouts, while 1.30 ± 0.20 N (average force) and 1.92 ± 0.36 N (maximum force) had to be applied to the sample containing 4% (*w*/*w*) Brussels sprouts (Figure 7a,b). No statistically significant differences between positive control and the samples in both the average as well as the maximum shear force were detected (*p* > 0.05).

The analysis of the mechanical properties did not reveal any significant differences between the positive controls and the sausage meat samples that contained 1.6 or 4.0% (*w*/*w*) Brussels sprouts. These findings indicate that the addition of freeze-dried Brussels sprouts did not influence the texture of the sausage meat samples. Thus, it can be assumed that no difference would be noticeable during the chewing process. Choe et al., however, described that phosphate-free samples were softer compared to phosphate-containing controls [25]. This deviating observation might result from the different amounts of dietary fibers in winter mushrooms and Brussels sprouts. Han and Bertram reported that dietary fibers could disrupt the formation of protein–water and protein–protein gel networks and subsequently decrease the gel strength of the meat product [36]. Thus, the use of Brussels sprouts, which contain fewer dietary fibers than winter mushrooms, resulted in a comparable texture to phosphate-containing meat products.

## 4. Conclusions

The present study revealed the beneficial properties of freeze-dried vegetables for their potential use as phosphate substitutes in sausage production. The addition of 1.6% freeze-dried Brussels sprouts had a similar effect on the WHC of sausage meat samples as the addition of 0.6% (*w*/*w*) phosphate in the positive control. Acceptance tests showed similar acceptance of sausage meat containing sweet corn and the positive control, while samples containing Brussels sprouts and Red Kuri squash received slightly lower acceptance values. This factor has to be considered in product development. None of the freeze-dried vegetables caused any significant changes in mechanical properties such as softness or tenderness. The color, however, was significantly altered because of the vegetables’ color.

The exchange of phosphate for freeze-dried vegetables may be a promising strategy in sausage production that offers an opportunity to progress towards the “clean-label” concept. The use of novel ingredients will be much easier to implement and less risky for the industry than investing in expensive new technologies. Further studies are now required to develop new sausage recipes that use freeze-dried vegetables as phosphate substitutes, but contain fat and spices to provide the expected taste, and to investigate the microbial stability of the new products.

## Figures and Tables

**Table 1:** pH Screening of different vegetables and fruits. Vegetable foods marked with an asterisk (*) have been used for further investigation.

Vegetable/Fruit	Plant Family	Fresh/Frozen/Dried	pH
Peas *	*Fabaceae*	frozen	7.25
Spinach *	*Amaranthaceae*	frozen	6.99
Red Kuri squash *	*Cucurbitaceae*	fresh	6.92
Sweet corn *	*Poaceae*	fresh	6.81
Avocado *	*Lauraceae*	fresh	6.80
Zucchini	*Cucurbitaceae*	fresh	6.54
Brussels sprouts *	*Brassicaceae*	frozen	6.45
Cassava	*Euphorbiaceae*	fresh	6.39
White cabbage	*Brassicaceae*	fresh	6.39
Broccoli	*Brassicaceae*	fresh	6.38
Cantaloupe	*Cucurbitaceae*	fresh	6.36
Beetroot	*Amaranthaceae*	fresh	6.17
Savoy cabbage	*Brassicaceae*	fresh	6.02
Celery root	*Apiaceae*	fresh	5.91
Sweet potato	*Convolvulaceae*	fresh	5.87
Date	*Arecaceae*	dried	5.70
Papaya (pulp/kernels) ^1^	*Caricaceae*	fresh	5.45/5.53

^1^ For papaya, pulp and kernels were analyzed separately.

**Table 2:** Values for labelled affective magnitude (LAM) determined by an untrained sensory panel for meat samples prepared with different amounts of freeze-dried Brussels sprouts, Red Kuri squash, or sweet corn and the positive control containing 0.6% commercial phosphate additive. Statistically significant differences between the positive control and the samples were evaluated. The levels of significance were * *p* ≤ 0.05; ** *p* ≤ 0.01; and not significant (ns) *p* > 0.05.

Additive	Added Conc. ^1^	Average LAM ^2^ Value				Minimum LAM ^2^ Value	Maximum LAM ^2^ Value
Brussels sprouts	4.0%	50.04	±	13.52	(*)	22.25	78.06
	1.6%	49.96	±	14.97	(*)	22.25	78.06
Red Kuri squash	4.0%	45.92	±	15.63	(**)	12.25	87.11
	1.6%	52.17	±	15.98	(ns)	22.25	78.06
Sweet corn	4.0%	57.53	±	14.91	(ns)	34.06	87.11
	1.6%	55.61	±	16.96	(ns)	22.25	87.11
Positive control		59.78	±	15.39		34.06	87.11

^1^ Conc., concentration; ^2^ LAM, labelled affective magnitude.

**Table 3:** Values for lightness (L*), red/green coordinates (a*), and yellow/blue coordinates (b*) determined by a spectrophotometer for sausage meat samples prepared with different amounts of freeze-dried Brussels sprouts, Red Kuri squash, or sweet corn and the positive control containing 0.6% commercial phosphate additive. Statistically significant differences between the positive control and the samples were evaluated. The levels of significance were * *p* ≤ 0.05; ** *p* ≤ 0.01; *** *p* ≤ 0.001; and not significant (ns) *p* > 0.05.

Additive	Added Conc. ^1^	L* (D65)	a* (D65)	b* (D65)
Brussels sprouts	4.0%	76.44 ± 0.88 (***)	1.27 ± 0.15 (**)	19.37 ± 0.36 (**)
	3.4%	75.73 ± 1.53 (***)	1.46 ± 0.35 (***)	18.54 ± 0.79 (**)
	2.8%	75.95 ± 1.62 (***)	1.56 ± 0.19 (***)	18.48 ± 0.30 (**)
	2.2%	75.98 ± 1.83 (***)	1.62 ± 0.28 (***)	18.39 ± 0.83 (**)
	1.6%	76.29 ± 1.72 (***)	1.48 ± 0.28 (***)	17.68 ± 0.64 (ns)
	1.0%	76.80 ± 2.36 (**)	1.68 ± 0.38 (***)	17.01 ± 0.83 (ns)
Red Kuri squash	4.0%	75.94 ± 0.85 (***)	4.03 ± 0.71 (***)	24.80 ± 2.49 (***)
	3.4%	75.50 ± 1.35 (***)	3.90 ± 0.47 (***)	24.10 ± 1.77 (***)
	2.8%	75.60 ± 1.29 (***)	3.52 ± 0.70 (***)	22.80 ± 2.48 (***)
	2.2%	75.97 ± 1.34 (***)	3.25 ± 0.78 (***)	21.75 ± 1.66 (***)
	1.6%	76.58 ± 1.37 (**)	2.84 ± 0.63 (***)	19.98 ± 1.15 (***)
	1.0%	76.60 ± 1.49 (**)	2.50 ± 0.46 (***)	19.17 ± 1.04 (ns)
Sweet corn	4.0%	75.86 ± 2.30 (**)	2.71 ± 0.53 (***)	21.12 ± 1.14 (***)
	3.4%	77.06 ± 2.03 (*)	2.42 ± 0.43 (***)	20.59 ± 1.02 (***)
	2.8%	76.27 ± 1.61 (***)	2.61 ± 0.28 (***)	20.07 ± 0.91 (***)
	2.2%	76.75 ± 2.87 (*)	2.29 ± 0.53 (***)	19.07 ± 0.89 (*)
	1.6%	76.31 ± 2.13 (**)	2.35 ± 0.55 (***)	18.82 ± 1.06 (ns)
	1.0%	76.45 ± 2.23 (**)	2.10 ± 0.47 (***)	17.33 ± 0.56 (ns)
Positive control		78.91 ± 1.16	0.87 ± 0.39	17.29 ± 1.29

^1^ conc., concentration.

## Data Availability

The data presented in this study are available on request from the corresponding author.

## Supplementary Materials

The following supporting information can be downloaded at: https://www.mdpi.com/article/10.3390/foods12101960/s1, Table S1 Sample formulations for the preparation of sausage meat samples as described in Section 2.5. Table S2 Attributes that were provided to panelists to indicate their score of acceptance and their translation into LAM values. Table S3 pH value in sausage meat containing 4–1% (*w*/*w*) freeze-dried Brussels sprouts, Red Kuri squash, or sweet corn (mean ± standard deviation). Figure S1 (a) Resulting curve for indentation test with marked loading, holding, and unloading phase for a sample containing 4.0% (*w*/*w*) Brussels sprouts. (b) Calculated linear regression (red) in loading phase to determine the slope *k* that is needed for the calculation of the effective modulus. Figure S2 Typical cyclic compression curves are shown for a sample containing 4.0% (*w*/*w*) Brussels sprouts. Three cycles were analyzed: black (first cycle), blue (second cycle), and red (third cycle). Figure S3 Typical curve for Warner–Bratzler shear force measurement for a sample containing 4.0% (*w*/*w*) Brussels sprouts.

## References

[1] EU (2008). Regulation (EC) Nr. 1333/2008 of the European Parliament and of the Council of 16 December 2008 on food additives. Off. J. Eur. Union.

[2] Ritz E., Hahn K., Ketteler M., Kuhlmann M.K., Mann J. (2012). Phosphate additives in food--a health risk. Dtsch. Arztebl. Int..

[3] Huang M.S., Sage A.P., Lu J., Demer L.L., Tintut Y. (2008). Phosphate and pyrophosphate mediate PKA-induced vascular cell calcification. Biochem. Biophys. Res. Commun..

[4] Giachelli C.M. (2009). The emerging role of phosphate in vascular calcification. Kidney Int..

[5] Onufrak S.J., Bellasi A., Shaw L.J., Herzog C.A., Cardarelli F., Wilson P.W., Vaccarino V., Raggi P. (2008). Phosphorus levels are associated with subclinical atherosclerosis in the general population. Atherosclerosis.

[6] Cancela A.L., Santos R.D., Titan S.M., Goldenstein P.T., Rochitte C.E., Lemos P.A., dos Reis L.M., Graciolli F.G., Jorgetti V., Moyses R.M. (2012). Phosphorus is associated with coronary artery disease in patients with preserved renal function. PLoS ONE.

[7] Chambers E., Chambers E., Castro M. (2018). What is “natural”? Consumer responses to selected ingredients. Foods.

[8] Asioli D., Aschemann-Witzel J., Caputo V., Vecchio R., Annunziata A., Naes T., Varela P. (2017). Making sense of the “clean label” trends: A review of consumer food choice behavior and discussion of industry implications. Food Res. Int..

[9] Bright B.K. Understanding emerging trends, ingredients and nutrients in processed meats to tell your best brand story. Proceedings of the Annual Meat Conference.

[10] Cegiełka A. (2020). “Clean label” as one of the leading trends in the meat industry in the world and in Poland—A review. Rocz. Państwowego Zakładu Hig..

[11] Lawrie R.A., Ledward D.A. (2006). Lawrie’s Meat Science.

[12] Cheng Q., Sun D.-W. (2008). Factors Affecting the Water Holding Capacity of Red Meat Products: A Review of Recent Research Advances. Crit. Rev. Food Sci. Nutr..

[13] Offer G., Trinick J. (1983). On the mechanism of water holding in meat: The swelling and shrinking of myofibrils. Meat Sci..

[14] Seman D.L., Olson D.G., Mandigo R.W. (1980). Effect of reduction and partial replacement of sodium on Bologna characteristics and acceptability. J. Food Sci..

[15] Trout G.R., Schmidt G.R. (1984). Effect of phosphate type and concentration, salt level and method of preparation on binding in restructured beef rolls. J. Food Sci..

[16] Brewer M.S., Dikeman M., Devine C. (2014). Chemical and physical characteristics of meat. Water holding capacity. Encyclopedia of Meat Sciences.

[17] Borch E., Kant-Muermans M.-L., Blixt Y. (1996). Bacterial spoilage of meat and cured meat products. Int. J. Food Microbiol..

[18] Delgado-Pando G., Ekonomou S.I., Stratakos A.C., Pintado T. (2021). Clean label alternatives in meat products. Foods.

[19] Thangavelu K.P., Kerry J.P., Tiwari B.K., McDonnell C.K. (2019). Novel processing technologies and ingredient strategies for the reduction of phosphate additives in processed meat. Trends Food Sci. Technol..

[20] Genccelep H., Saricaoglu F.T., Anil M., Agar B., Turhan S. (2015). The effect of starch modification and concentration on steady-state and dynamic rheology of meat emulsions. Food Hydrocoll..

[21] Zhou L., Zhang W., Wang J. (2022). Recent advances in the study of modified cellulose in meat products: Modification method of cellulose, meat quality improvement and safety concern. Trends Food Sci. Technol..

[22] Powell M.J., Sebranek J.G., Prusa K.J., Tarté R. (2019). Evaluation of citrus fiber as a natural replacer of sodium phosphate in alternatively-cured all-pork Bologna sausage. Meat Sci..

[23] Magalhães I.M.C., Paglarini C.D.S., Vidal V.A.S., Pollonio M.A.R. (2020). Bamboo fiber improves the functional properties of reduced salt and phosphate-free Bologna sausage. J. Food Process. Preserv..

[24] Petracci M., Laghi L., Rocculi P., Rimini S., Panarese V., Cremonini M.A., Cavani C. (2012). The use of sodium bicarbonate for marination of broiler breast meat. Poult. Sci..

[25] Choe J., Lee J., Jo K., Jo C., Song M., Jung S. (2018). Application of winter mushroom powder as an alternative to phosphates in emulsion-type sausages. Meat Sci..

[26] Nunez S.M., Cardenas C., Pinto M., Valencia P., Cataldo P., Guzman F., Almonacid S. (2020). Bovine skin gelatin hydrolysates as potential substitutes for polyphosphates: The role of degree of hydrolysis and pH on water-holding capacity. J. Food Sci..

[27] Parsons A.N., VanOverbeke D.L., Goad C.L., Mireles DeWitt C.A. (2011). Retail display evaluation of steaks from select beef strip loins injected with a brine containing 1% ammonium hydroxide. Part 2: Cook yield, tenderness, and sensory attributes. J. Food Sci..

[28] Belitz H.-D., Grosch W., Schieberle P. (2009). Food Chemistry.

[29] Souci S.W., Fachmann W., Kraut H. (2016). Food Composition and Nutrition Tables.

[30] Kemp S.E., Hollowood T., Hort J. (2009). Sensory Evaluation: A Practical Handbook.

[31] Schutz H.G., Cardello A.V. (2001). A labeled affective magnitude (LAM) scale for assessing food liking/disliking. J. Sens. Stud..

[32] Greene J.L., Bratka K.J., Drake M.A., Sanders T.H. (2006). Effectiveness of category and line scales to characterize consumer perception of fruity fermented flavor in peanuts. J. Sens. Stud..

[33] Lawless H.T., Popper R., Kroll B.J. (2010). A comparison of the labeled magnitude (LAM) scale, an 11-point category scale and the traditional 9-point hedonic scale. Food Qual. Prefer..

[34] Budday S., Nay R., de Rooij R., Steinmann P., Wyrobek T., Ovaert T.C., Kuhl E. (2015). Mechanical properties of gray and white matter brain tissue by indentation. J. Mech. Behav. Biomed. Mater..

[35] Uzlaşır T., Aktaş N.i., Gerçekaslan K.E. (2020). Pumpkin seed oil as a partial animal fat replacer in Bologna-type sausages. Food Sci. Anim. Resour..

[36] Han M., Bertram H.C. (2017). Designing healthier comminuted meat products: Effect of dietary fibers on water distribution and texture of a fat-reduced meat model system. Meat Sci..

[37] Verma A.K., Sharma B.D., Banerjee R. (2010). Effect of sodium chloride replacement and apple pulp inclusion on the physico-chemical, textural and sensory properties of low fat chicken nuggets. LWT Food Sci. Technol..

[38] Ahmad S.S., Khalid M., Younis K. (2020). Interaction study of dietary fibers (pectin and cellulose) with meat proteins using bioinformatics analysis: An In-Silico study. LWT.

[39] Fenwick G.R., Griffiths N.M. (1981). The identification of the goitrogen (-)5-vinyloxazolidine-2-thione (goitrin), as a bitter principle of cooked brussels sprouts (*Brassica oleracea* L. var. *gemmifera*). Z. Lebensm. Unters. Forsch..

[40] Beck T.K., Jensen S., Bjoern G.K., Kidmose U. (2014). The masking effect of sucrose on perception of bitter compounds in Brassica vegetables. J. Sens. Stud..

