# Supplementary material for: Dried Vegetables as Potential Clean-Label Phosphate Substitutes in Cooked Sausage Meat
Source: Foods. 2023 May 11;12(10):1960. doi: 10.3390/foods12101960 (PMC10216979; doi:10.3390/foods12101960)
Supplement: Supplementary file 1 [file foods-12-01960-s001.zip › foods-2249620-supplementary.pdf]

Supplementary Materials

Dried Vegetables as Potential Clean-Label Phosphate Substitutes in Cooked Sausage Meat

Ingrid Weigel [1], Sarah Nistler [2], Rohtraud Pichner [3], Silvia Budday [2] and Sabrina Gensberger-Reigl [1,*]

[1] Food Chemistry, Department of Chemistry and Pharmacy, Faculty of Sciences, Friedrich-Alexander-Universität Erlangen-Nürnberg (FAU), Nikolaus-Fiebiger-Str. 10, 91058 Erlangen, Germany

[2] Institute of Applied Mechanics, Department of Mechanical Engineering, Faculty of Engineering, Friedrich-Alexander-Universität Erlangen-Nürnberg (FAU), Egerlandstr. 5, 91058 Erlangen, Germany

[3] Department of Nutritional, Food and Consumer Sciences, University of Applied Sciences, Leipziger Str. 123, 36037 Fulda, Germany

* Correspondence: sabrina.gensberger@fau.de; Tel.: +49-9131-8565600

Table S1 Sample formulations for the preparation of sausage meat samples as described in section 2.5.

Sample	Minced meat (g)	Sodium chloride solution (mL)[1]	Phosphates solution (mL)[2]	Freeze-dried vegetables (mg)	Ice-cold water (mL)
**Negative control**	5	1	-	-	4
**Positive control**	5	1	1	-	3
**4.0%**	5	1	-	200	4
**3.4%**	5	1	-	170	4
**2.8%**	5	1	-	140	4
**2.2%**	5	1	-	110	4
**1.6%**	5	1	-	80	4
**1.0%**	5	1	-	50	4

[1]concentration: 50 mg/mL
[2]concentration: 30 mg/mL

Table S2 Attributes that were provided to panelists to indicate their score of acceptance and their translation into LAM values.

Attribute	Value
Greatest imaginable liking	**100.00**
Like extremely	**87.11**
Like very much	**78.06**
Like moderately	**68.12**
Like slightly	**55.62**
Neither like or dislike	**50.00**
Dislike slightly	**44.69**
Dislike moderately	**34.06**
Dislike very much	**22.25**
Dislike extremely	**12.25**
Greatest imaginable dislike	**0.00**

Table S3 pH Value in sausage meat containing 4–1% (w/w) freeze-dried Brussels sprouts, Red Kuri squash, or sweet corn (mean ± standard deviation).

	**Brussels sprouts**		**Red Kuri squash**		**Sweet corn**	
	Mean pH	SD*	Mean pH	SD	Mean pH	SD
Negative control	5.79	±0.03	5.78	±0.05	5.79	±0.04
Positive control	5.88	±0.01	5.87	±0.03	5.88	±0.02
4.0%	5.81	±0.02	5.82	±0.03	5.84	±0.04
3.4%	5.81	±0.02	5.81	±0.04	5.83	±0.04
2.8%	5.81	±0.02	5.81	±0.04	5.82	±0.04
2.2%	5.80	±0.03	5.81	±0.04	5.82	±0.04
1.6%	5.80	±0.03	5.80	±0.03	5.81	±0.04
1.0%	5.80	±0.03	5.80	±0.04	5.80	±0.04

* SD, standard deviation

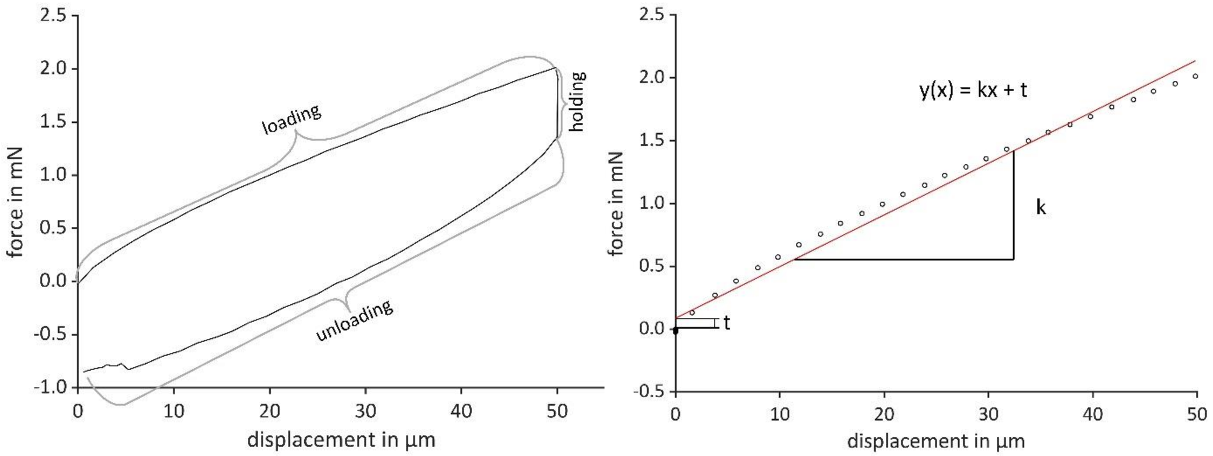

Figure S1 (a) Resulting curve for indentation test with marked loading-, holding- and unloading phase for a sample containing 4.0% (w/w) Brussels sprouts. (b) Calculated linear regression (red) in loading phase to determine the slope *k* that is needed for the calculation of the effective modulus.

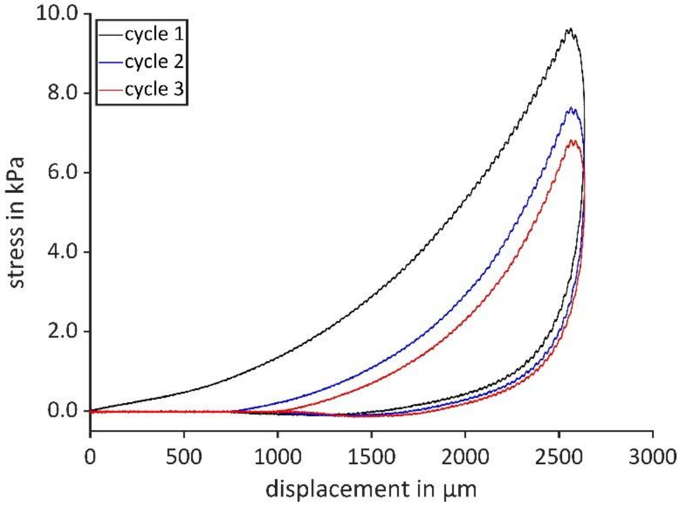

Figure S2 Typical cyclic compression curves for a sample containing 4.0% (w/w) Brussels sprouts. Three cycles were analyzed: black (first cycle), blue (second cycle), and red (third cycle).

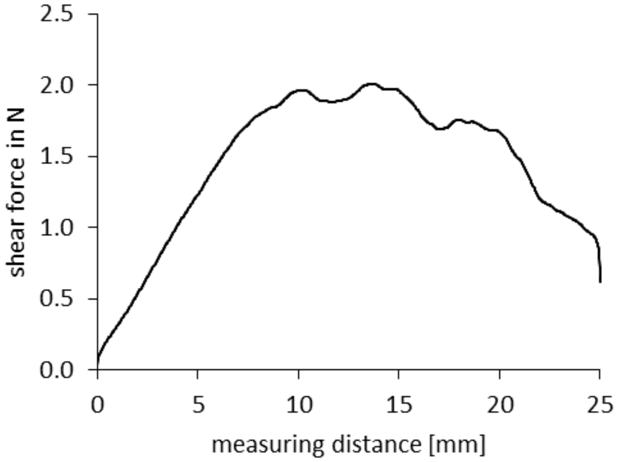

Figure S3 A typical curve for Warner Bratzler's shear force measurement for a sample containing 4.0% (w/w) Brussels sprouts.
